# Anastomotic Leak after Bariatric Surgery from a Critical Care Perspective: A Lesson Shared

**DOI:** 10.21315/mjms2018.25.5.15

**Published:** 2018-10-30

**Authors:** Wan Fadzlina Wan Muhd Shukeri, Mohd Hasyizan Hassan, Wan Mohd Nazaruddin Wan Hassan, Rhendra Hardy Mohamad Zaini

**Affiliations:** Department of Anaesthesiology and Critical Care, School of Medical Sciences, Universiti Sains Malaysia, 16150 Kubang Kerian, Kelantan, Malaysia

**Keywords:** anastomotic leak, bariatric surgery, critical care

## Abstract

Anastomotic leak after bariatric surgery is a rare complication with a recent prevalence ranging from 0.8% to 1.5%. The complication nevertheless can result in morbidity and even mortality. The purpose of this paper is to present a patient who suffered from an anastomotic leak presenting 2 days after laparoscopic sleeve gastrectomy in our intensive care unit. Review of the current literature regarding this complication from critical care perspective is also attempted.

## Introduction

Hospital Universiti Sains Malaysia is still on a learning curve in bariatric surgery. We wish to share our first experience dealing with an anastomotic leak after a laparoscopic sleeve gastrectomy from a critical care perspective. Anastomotic leak after bariatric surgery is a rare complication in established centres, with a recent prevalence ranging from 0.8% to 1.5% (1).

## Case Report

A 55-year-old female patient presented to our intensive care unit (ICU) after laparoscopic gastric bypass surgery. She had underlying morbid obesity, with a body mass index of 53.6 kg/m^2^, hypertension and clinically probable obstructive sleep apnoea. Intra-operatively, the surgery was protracted to about eight hours due to technical difficulties. Due to her underlying comorbidities and prolonged surgery, she was admitted to the ICU for post-operative observation and remained intubated. Following extubation on post-operative day 2, she developed persistent tachycardia and hypoxemic respiratory failure and was commenced on noninvasive ventilation (NIV). A few hours later, she developed epigastric pain. An anastomotic leak was suspected, which was confirmed by endoscopic findings. Subsequently, urgent surgical repair of the leak was carried out. She recovered over a course of 5 days after the re-surgery and was discharged to the ward.

## Discussion

When our patient had persistent tachycardia and respiratory failure during the post-operative period, the possibility of an anastomotic leak did not come to our minds. High on our list of differential diagnoses were pulmonary embolism and decompensated OSA. Hamilton et al. found these signs (i.e., tachycardia and respiratory failure) to be the two most common presenting manifestations of anastomotic leak in a recent review of 210 consecutive laparoscopic gastric bypass patients (2). In addition, one may raise a concern regarding the use of NIV after gastrointestinal anastomosis, which theoretically can cause anastomotic leak by increasing the intraluminal gastric pressure (1). In recent years, this modality has emerged as a useful approach to improve oxygenation in OSA patients who develop hypoxaemia in the immediate post-operative period of bariatric surgery. Most of the currently available literature has reported favourable outcomes from the usage of NIV in post-operative bariatric surgery (1, 3, 4).

## Conclusion

We learned that persistent tachycardia, failure of progression to extubation and/or sudden respiratory decompensation in the immediate post-operative period of bariatric surgery should raise suspicion of intraabdominal pathology. In the presence of these signs, a diagnosis of anastomotic leak should be considered, in addition to other common causes of post-operative respiratory decline. The use of NIV to treat hypoxemia in OSA patients after gastrointestinal anastomosis should not be contra-indicated, as long as the upper limit of airway pressure is observed.
